# Examining co-patterns of depression and alcohol misuse in emerging adults following university graduation

**DOI:** 10.1016/j.abrep.2018.06.002

**Published:** 2018-06-12

**Authors:** Jona R. Frohlich, Karli K. Rapinda, Roisin M. O'Connor, Matthew T. Keough

**Affiliations:** a Department of Psychology, University of Manitoba, 190 Dysart Rd., Winnipeg, MB R3T 2N2, Canada; b Department of Psychology, Concordia University, 7141 Sherbrooke St. West, Montreal, QC H4B 1R6, Canada

**Keywords:** Alcohol misuse, Depression, Emerging adults, High-risk individuals

## Abstract

Depression and alcohol use disorders are highly comorbid. Typically, alcohol use peaks in emerging adulthood (e.g., during university), and many people also develop depression at this time. Self-medication theory predicts that depressed emerging adults drink to reduce negative emotions. While research shows that depression predicts alcohol use and related problems in undergraduates, far less is known about the continuity of this association after university. Most emerging adults “mature out” of heavy drinking; however, some do not and go on to develop an alcohol use disorder. Depressed emerging adults may continue to drink heavily to cope with the stressful (e.g., remaining unemployed) transition out of university. Accordingly, using parallel process latent class growth modelling, we aimed to distinguish high- from low-risk groups of individuals based on joint patterns of depression and alcohol misuse following university graduation. Participants (*N* = 123) completed self-reports at three-month intervals for the year post-graduation. Results supported four classes: class 1: *low stable depression and low decreasing alcohol misuse* (*n* = 52), class 2: *moderate stable depression and moderate stable alcohol misuse* (*n* = 35), class 3: *high stable depression and low stable alcohol misuse* (*n* = 29), and class 4: *high stable depression and high stable alcohol misuse* (*n* = 8). Our findings show that the co-development of depression and alcohol misuse after university is not uniform. Most emerging adults in our sample continued to struggle with significant depressive symptoms after university, though only two classes continued to drink at moderate (class 2) and high (class 4) risk levels.

## Highlights

• Co-patterns of depression and alcohol misuse were examined in emerging adults.
• Four distinct classes were supported.
• Co-patterns of depression and alcohol misuse were not uniform.
• Some continue to struggle with alcohol misuse and depression after graduation.

## 1 Introduction

Depression and alcohol misuse are highly co-occurring problems (Grant et al., 2004). It is one of the most frequent mental health comorbidities in the general population (Conway, Compton, Stinson, & Grant, 2006), with about one-third of people with a depressive disorder also meeting criteria for an alcohol use disorder (AUD; Davis, Uezato, Newell, & Frazier, 2008). It is critical to examine the comorbidity between depression and alcohol misuse, as people who suffer from both (relative to those with just one of these problems) present with greater clinical severity, have poorer treatment responses, and are more likely to relapse (Baker et al., 2007; Lubman, Allen, Rogers, Cementon, & Bonomo, 2007). Thus, researchers should conduct studies to better understand the pathways of comorbid depression and alcohol misuse.

Emerging adulthood (ages 18 to 25) is a period of new freedom (Arnett, 2005). Most emerging adults in Canada attend some form of post-secondary school (Shaienks, Gluszynski, & Bayard, 2008) and for many, this is often their first time living independently from their parents. During this time, there is a normative developmental peak in alcohol use and other risky behaviours, and as a result, emerging adults experience diverse and severe alcohol problems (Arnett, 2005; Johnston, O'Malley, Bachman, & Schulenberg, 2007). The prevalence of alcohol use disorders (AUDs) is highest in emerging adulthood relative to other developmental periods (Adlaf, Demers, & Gliksman, 2005; NIAAA, 2015). Not only does alcohol misuse (i.e., heavy drinking, alcohol-related problems) peak during this time, but there is also a rapid increase in the number of people suffering from depression symptoms (Bayram & Bilgel, 2008). Nearly 30% of emerging adults report struggling with depressive symptoms (Ibrahim, Kelly, Adams, & Glazebrook, 2013), and depression relates to drinking problems in this population (Grothues et al., 2008).

Self-medication theory provides a framework to understand why depression and alcohol misuse co-occurs in emerging adulthood. The self-medication hypothesis posits that people drink alcohol to numb painful emotions (Khantzian, 1997). Over time, emerging adults with depression learn that alcohol helps them cope with negative feelings, and this increases risk for alcohol problems. Consistent with theory, the literature shows that emerging adults with depression endorse coping motives for drinking (Grant, Stewart, & Mohr, 2009), which in turn increase risk for alcohol problems. However, emerging adults who drink to cope do not seem to be drinking heavier than their peers who drink for other reasons (e.g., to feel positive emotions; Kuntsche, Knibbe, Gmel, & Engels, 2005). One potential explanation for this is that many people drink heavily in university – a context where heavy drinking is accepted and even encouraged – therefore, it may be difficult to tease apart co-patterns of depression and drinking during this time.

Most emerging adults “mature out” of heavy drinking after graduating from university; however, some people do not and are the ones at high risk to develop serious alcohol problems later in life. Thus, these individuals are at a greater risk for alcohol misuse compared to those who “mature out.” The transition out of university may be when depression-related drinking diverges from the drinking patterns of non-depressed emerging adults. This is a stressful and potentially defeating transition (Wendlandt & Rochlen, 2008), and these people may feel lost, experience difficulty finding work, remain unemployed, and suffer from increased levels of negative affect (Keough & O'Connor, 2016). Consequently, depressed emerging adults may continue to drink heavily to cope during this tough time and experience more alcohol-related problems as a result, while their non-depressed peers mature out. Therefore, the transition out of university may be a particularly critical time to examine the co-development of depression and alcohol misuse. This has implications for understanding how depressive pathways to drinking problems unfold from emerging to full adulthood.

While the relationship between depression and alcohol misuse in undergraduates is well supported (e.g., Mushquash et al., 2013), research on patterns of misuse after university is limited. We will be among the first to examine the co-development of these constructs during the critical, potentially defeating transition out of university. If we can identify meaningful transition points in emerging adulthood, there may be opportunities to intervene early and thus improve their overall mood and well being in the future. Therefore, we used parallel process latent class growth modelling (LCGM) to identify potential subgroups based on joint trajectories of depression and alcohol misuse following university graduation. The overarching hypothesis was that using parallel process LCGM, we would be able to distinguish high from low risk classes. While we did not predict the specific number of co-development classes, we hypothesized that at least one group would emerge as high risk (i.e., high levels of depression and alcohol misuse), given the associations between depression and alcohol problems (DeVido & Weiss, 2012).

## 2 Method

### 2.1 Participants and procedure

Undergraduates were recruited from two English-speaking universities in Montreal (*N* = 123; *Mage* = 23.18, *SDage* = 2.17; 71% women). Participants were recruited using a snowball sampling method, through means such as flyers, online research participant pools, and by providing information sessions to university classes. In order be eligible to participate, students had to: a) be in their graduating year of undergraduate studies; b) not have taken more than one-term (i.e., four consecutive months) off from school (excluding summer); c) be a full-time student; and d) be fluent in English. Eligible participants completed hour-long online assessments at baseline (one-to-two months before graduation) and at three-month intervals post graduate for one year (five measurements total). The goal of the initial assessment was to obtain baseline levels of each construct before participants left university. All participants were given a maximum of one month to complete the measures for a given time point assessment. In the initial sample, 61% of students were Caucasian and minority ethnicities represented were East Asian, South-East Asian, and Pacific Islander (9%); Middle Eastern, North African, and Central Asian (9%); Hispanic (6%); Black (4%); South Asian (3%); Aboriginal (1%); and 7% reported “other.” Participants were compensated with $15 CAD per survey with a potential to receive $25 CAD for completing surveys at all time points ($100 max compensation). All participants gave informed consent to participate in this research study. The Ethics Review Board at Concordia University approved study procedures. Data was taken from a larger study examining patterns of maturing out among undergraduate students (Keough & O'Connor, 2016).

Of the initial sample, 85% of participants completed the three-month assessment (*n* = 101), 74% completed the six-month assessment (*n* = 88), 70% completed the nine-month assessment (*n* = 82), and 62% completed the final one-year assessment (*n* = 74). Of participants who completed surveys at all time points (*n* = 69), employment status at one-year was as follows: 52% full time, 32% part-time, and 16% unemployed. Also, at one-year follow-up, 71% of participants were not enrolled in any postsecondary education, 23% were in a graduate program, and a small minority (6%) returned to complete part-time undergraduate studies.

### 2.2 Measures

#### 2.2.1 The Alcohol Use Disorders Identification Test

The Alcohol Use Disorders Identification Test (AUDIT; Saunders, Aasland, Barbor, de la Fuente, & Grant, 1993) uses 10 items to provide an assessment of alcohol misuse, which includes measurement of alcohol use and related problems (e.g., “*How often during the last year have you found that you were not able to stop drinking once you had started?*”) The AUDIT was administered at all time points and was used as the primary measure to assess maturing out of alcohol misuse. Participants responded to items on response scales, ranging from 0 (*never*) to 4 (*four or more times a week*) for items 1–8, and from 0 (*no*) to 2 (*yes, during the last year*) for items 9 and 10. Total sum scores were used. The AUDIT has been shown to have very good test-retest reliability (*r* = 0.84) and adequate internal consistency (α = 0.76).

#### 2.2.2 The Center for Epidemiological Studies Depression Scale

The Center for Epidemiological Studies Depression Scale (CES-D; Radloff, 1977) includes 20 items and measures depressive symptoms in the past week (e.g., “*I felt that everything I did was an effort*”). The CES-D was given at all time points and was used as the primary measure of depressive symptoms. Participants responded to items on scales ranging from 0 (*rarely or never*) to 3 (*most or all of the time*). Total scores were used. The CES-D has been shown to have high internal consistency (α = 0.85–0.90) and moderate test-retest reliability (*r* = 0.40; Radloff, 1977).

### 2.3 Data analysis plan

Prior to running the parallel process LCGM, descriptive statistics and correlations were inspected. A missing data analysis was then conducted to examine any potential baseline group differences between participants with complete versus those with incomplete. Next, using Mplus version 8.0, a parallel process LCGM analysis was conducted to examine the joint trajectories (or co-development) of depression and alcohol misuse among emerging adults during their transition year out of university. Parallel process LCGM analysis allows for the identification of unobserved distinct groups of people who have similar developmental trajectories (Muthen & Muthen, 2000). In our study, we were interested in the number of subgroups (or classes) based on initial levels and changes in *both* alcohol misuse *and* depression symptoms.

Consistent with recommendations (Jung & Wickrama, 2008), we examined models with one to five classes. To determine the best fitting class solution, we used the Bayesian Information Criterion (BIC) and entropy values (Jung & Wickrama, 2008).^1^ Low BIC values indicate better model fit and a difference of 10 or more indicates superior fit in the model with the lower BIC value (Raftery, 1995). Entropy values reflect how well cases are classified into one group only and values closer to 1.0 indicate better classification quality (Ram & Grimm, 2009; Wang & Bodner, 2007). To avoid issues related to modelling over-fitting, class sizes of smaller than 5% of the total sample size were not permitted. Models were estimated with 800 random starts to avoid any issues related to local maxima.

## 3 Results

### 3.1 Descriptive statistics, correlations, and missing data analysis

Descriptive statistics and correlations are presented in Table 1. Mean levels of depression were above CES-D cut-offs for risk of depression (≥16; Radloff, 1977) across all time points. In contrast, mean levels of alcohol misuse were below the established cut-off score on the AUDIT for risky drinking (≥8; Saunders et al., 1993) across time. Bivariate associations show high stability of both depression and alcohol misuse over time. Finally, there were some significant positive correlations among depression and alcohol misuse variables across time, but as seen in Table 1, these were not always observed. Nevertheless, these correlations provide support for examining joint class trajectories in this sample of emerging adults.

**Table 1:** Descriptives statistics and bivariate correlations.

	1	2	3	4	5	6	7	8	9	10
1. T1 alcohol misuse (AUDIT)	–	0.69⁎⁎	0.67⁎⁎	0.68⁎⁎	0.64⁎⁎	0.17	0.06	0.23⁎	0.24⁎	0.15
2. T2 alcohol misuse (AUDIT)		–	0.65⁎⁎	0.62⁎⁎	0.73⁎⁎	0.12	0.09	0.13	0.22⁎	0.20⁎
3. T3 alcohol misuse (AUDIT)			–	0.75⁎⁎	0.77⁎⁎	0.17	0.09	0.18	0.19	0.11
4. T4 alcohol misuse (AUDIT)				–	0.67⁎⁎	0.01	0.09	0.09	0.22⁎	−0.06
5. T5 alcohol misuse (AUDIT)					–	0.21	0.04	0.08	0.14	0.27⁎
6. T1 depression (CES-D)						–	0.51⁎⁎	0.64⁎⁎	0.29⁎⁎	0.45⁎⁎
7. T2 depression (CES-D)							–	0.65⁎⁎	0.51⁎⁎	0.64⁎⁎
8. T3 depression (CES-D)								–	0.55⁎⁎	0.42⁎⁎
9. T4 depression (CES-D)									–	0.45⁎⁎
10. T5 depression (CES-D)										–
*Mean*	7.09	5.53	5.25	4.89	4.61	22.13	22.61	21.60	21.38	21.90
*SD*	5.05	4.57	4.48	4.30	3.97	9.11	9.85	9.32	9.68	10.28
Skewness	0.87	1.54	1.24	1.63	1.49	0.80	0.63	0.84	0.90	0.51
Kurtosis	0.17	2.62	1.09	2.74	2.58	0.21	−0.29	0.69	0.73	−0.43

Note. AUDIT = Alcohol Use Disorders Identification Test; CES-D = The Center for Epidemiological Studies Depression Scale.

⁎ *p* < .05.

⁎⁎ *p* < .01.

Missing data analyses indicated that participants who completed all five time points (*n* = 69) did not statistically differ from those with incomplete data (*n* = 54) in baseline depressive symptoms (*t*(121) = 0.438, *p* = .66, *d* = 0.088), or alcohol misuse (*t*(121) = 1.856, *p* = .07, *d* = 0.340), and effect sizes were also small in magnitude. The binary missingness variable was also uncorrelated with gender (*r* = 0.12, *p* = .19) and age (*r* = −0.05, *p* = .58). Given these lack of differences, we assume that data are at least missing at random (Enders, 2010). Multiple imputation was used to account for missing data in statistical models.

### 3.2 Determining the number of joint depression-alcohol misuse classes

**Fig. 1:**
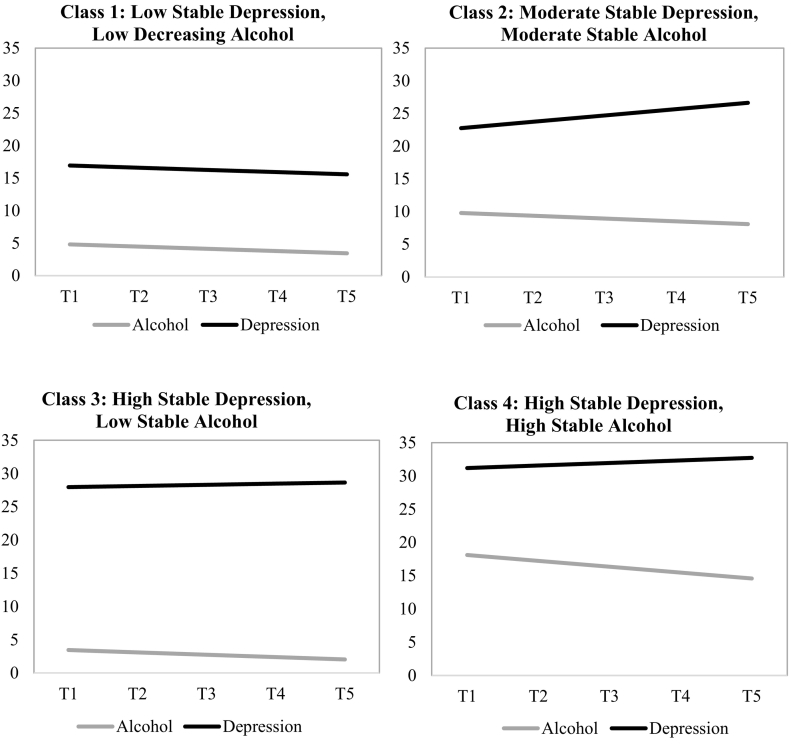
Co-development of depression and alcohol misuse among the four classes.

Fit information for models with one-to-five classes is presented in Table 2. As can be observed, BIC values steadily decreased moving from one-to-five class solutions. The entropy values for all models were well above 0.80, suggesting good classification quality overall. The model with five classes had a very small group (4.3% of the sample), and thus, the model with four classes was retained (see Table 3). The first “normative” class was the largest group, with low stable depression and low decreasing alcohol misuse over time (*n* = 52) (see Fig. 1). The second class was defined by moderate stable depression and moderate stable alcohol misuse (*n* = 35) over the year. The third class was defined by high stable depression, but low stable alcohol misuse over the one-year post university (*n* = 29). Finally, high levels of both depression and alcohol misuse characterized the fourth class over time (*n* = 8) – suggesting that this was the “high risk” group.

**Table 2:** Fit indices for one-to-five latent class growth models (*N* = 123).

Number of classes	Fit statistics		Smallest group (%)
	BIC	Entropy	
1-Class	7863.91	N/A	N/A
2-Class	7797.77	0.922	26.7
3-Class	7673.51	0.931	5.6
**4-Class**	**7595.91**	**0.890**	**6.3**
5-Class	7559.36	0.893	4.2

Note. BIC = Bayesian Information Criterion; bold print indicates the best-fit statistic across the five models.

**Table 3:** Parameter estimates for parallel process latent class growth analysis.

Class		Depression	Alcohol misuse
1 Low stable depression, low decreasing alcohol misuse (*n* = 52)	Intercept	**16.95** (*p* < .001)	**4.81** (*p* < .001)
	95% CI	[14.96, 18.93]	[3.77, 5.85]
	Slope	−0.34 (*p* = .545)	**−0.34** (*p* = .045)
	95% CI	[−1.44, 0.76]	[−0.68, −0.01]
2 Moderate stable depression, moderate stable alcohol misuse (*n* = 35)	Intercept	**22.76** (*p* < .001)	**9.79** (*p* < .001)
	95% CI	[17.61, 27.91]	[8.37, 11.21]
	Slope	0.97 (*p* = .097)	−0.43 (*p* = .143)
	95% CI	[−0.18, 2.12]	[−0.99, 0.15]
3 High stable depression, low stable alcohol misuse (*n* = 29)	Intercept	**27.96** (*p* < .001)	**3.449** (*p* < .001)
	95% CI	[24.69, 31.32]	[2.11, 4.79]
	Slope	0.17 (*p* = .764)	−0.35 (*p* = .117)
	95% CI	[−0.96, 1.30]	[−0.80, 0.09]
4 High stable depression, high stable alcohol misuse (*n* = 8)	Intercept	**31.19** (*p* < .001)	**18.12** (*p* < .001)
	95% CI	[23.53, 38.85]	[14.84, 21.39]
	Slope	0.38 (*p* = .38)	−0.88 (*p* = .06)
	95% CI	[−2.85, 3.61]	[−1.79, 0.03]

Bold indicates statistically significant values.

## 4 Discussion

The goal of this study was to examine joint trajectories of alcohol misuse and depression among emerging adults in the year following university graduation. We observed differing co-patterns of alcohol misuse and depression among individuals transitioning out of university. Results revealed that individuals fell into four classes. The largest class was comprised of individuals who had relatively low (based on the sample) stable levels of depression and low decreasing alcohol misuse over the year post university. This “low risk” class likely reflects normative maturing out of alcohol misuse. The second class was “moderate risk” and included emerging adults who had moderate and stable co-occurring depressive symptoms and alcohol misuse. The third class was characterized by relatively high stable levels of depressive, and low stable alcohol misuse over the year post university. Finally, the fourth “high risk” class had emerging adults with the highest levels of pre-graduation depression and alcohol misuse (relative to the other classes), which both remained stable over the year after university graduation. Overall, results show that the co-development of depression and alcohol misuse after university is not uniform.

Our results illustrate that the largest class of emerging adults reduced alcohol misuse during the year following university graduation, as would be expected based on the maturing out literature (Littlefield, Sher, Wood, & Watson, 2009). While this class had the lowest scores on the CES-D (relative to other classes in our study), it should be noted that the overall stable depressive symptoms in this group were still at or above the cut-off score for risk of depression. This indicates that emerging adults, overall, are at risk for continued mood problems post university – though there was large variability in depression severity between groups. Two of the three remaining classes (Classes 2 and 4) struggled with stable co-occurring moderate and severe depression and alcohol misuse, respectively. This is consistent with previous research suggesting that coping-motivated drinking (Keough & O'Connor, 2016) and alcohol-related problems (Arnett, 2005; Johnston et al., 2007) are persistent challenges for highly depressed emerging adults, especially during the stressful life transition out of university. These findings lend support to the self-medication hypothesis, suggesting that individuals in these classes may have been continuing to drink heavily throughout the year in order to numb their persisting depressive symptoms, as well as experiencing resulting alcohol-related problems. As a result, depressed emerging adults may require assistance as they navigate this challenging time in their life. Targeted clinical support (pre-transition) could help to reduce the likelihood of alcohol misuse-depression continuity after university among emerging adults (Adlaf et al., 2005; Ibrahim et al., 2013).

Overall, our findings offer some support for the associations between depression and alcohol misuse in emerging adults as they transition out of full-time undergraduate studies. However, it should be noted that Class 3 was unexpected. Individuals in this class had high levels of depression (pre-graduation), which persisted over the year following university. They also had non-problematic stable levels of alcohol misuse. Based on the literature and the self-medication hypotheses, we would have expected these people to also be at risk for heavy drinking to cope with elevated mood problems – like we observed in Class 4. Due to small class sizes in our sample, we were unable to conduct follow-up analyses to examine potential variables that distinguish Class 3 (low risk for comorbidity) from Class 4 (high risk for comorbidity). One possibility is that individuals who had high stable levels of co-occurring depression and alcohol misuse (Class 4) also had additional problems (e.g., anxiety, negative urgency) that contributed to their continued high-risk drinking or alcohol related-problems. Future work with larger samples should replicate our longitudinal class analyses and clarify the factors that distinguish high from low risk groups.

The results have clinical implications. We identified that there is a small group of individuals (Class 4) who maintain high levels of depression and alcohol misuse in the year following graduation. There was also a larger group that struggled with moderate comorbidity over this transition (Class 2). Accordingly, this may be a critical time to intervene to reduce the likelihood of comorbid alcohol misuse and mood disorders among these individuals later in adulthood. Clinicians should emphasize the ongoing risks of depression-related drinking, especially during the transition out of university when life circumstances change. Clinicians could also work with “at risk” students during their final year to develop more effective coping strategies – with the hope that they will carry these forward into their lives post-university.

### 4.1 Limitations

The results should be interpreted in light of some limitations. First, we did not model the specific role transitions of participants (e.g., entering the work force, pursuing graduate studies, starting a family) throughout the year. However, a main reason why emerging adults mature out of drinking is that they transition in to roles that require a greater amount of responsibility, such as getting married, starting a family, and beginning full-time employment. Therefore, it is possible that participants would differ in regard to alcohol misuse and depression depending on their circumstances, especially given that patterns of heavy drinking would be inconsistent with succeeding in these new roles. Future research should expand on our results by including these role transitions in the model in order to determine the potentially different impact that each has on the co-development of alcohol misuse and depression. In addition to role transition, it also is possible that differences may exist between alcohol-related problems and alcohol use in regard to associations with depression. Thus, future research should examine patterns with both facets of alcohol misuse (i.e., problems and use) separately. Second, we had a fairly small sample size and attrition was high, which may have reduced our ability to detect more classes. However, missing data analyses revealed that those who did not complete all assessment points were missing at random. Nevertheless, our differentiation of high from low risk classes provides preliminary support for future model testing with larger samples. Furthermore, there was an overrepresentation of female participants. Examining the role of gender in future research would be beneficial, particularly given that patterns of alcohol misuse may differ post-university between men and women (Perkins, 1999). Finally, the trajectory of alcohol misuse and depression was only examined over one year, allowing us to capture only the initial stages of transition. It is realistic to assume that individuals experience changes and challenges for several years following university, which may influence depression and related alcohol misuse. Therefore, it would be beneficial to follow “at risk” individuals for many years after graduation, and later into adulthood.

## 5 Conclusion

A large body of research has examined patterns of alcohol misuse and depressive symptoms while emerging adults are in university. However, this study was one of the first to examine joint trajectories of these constructs in emerging adults as they transition out of undergraduate studies. In general, differing patterns of alcohol misuse and depressive comorbidity were observed. Findings suggest that individuals with elevated depression and alcohol misuse before graduation are likely to maintain these problems after university, and may develop comorbid mood and alcohol use disorders in the years to come. By identifying these critical transition points and patterns among struggling emerging adults, there may now be opportunities to intervene and thus improve their overall mood and well-being during this imperative point in their lives.

## Footnotes

1 The Lo-Mendell-Rubin likelihood ratio and the bootstrap likelihood ratio tests are also recommended to assess fit of latent class models (Jung & Wickrama, 2008; Nylund, Asparouhov, & Muthen, 2007). But, these tests are not available when imputed data are used.
